# Alternating hemiplegia of childhood misdiagnosed as hysteria: a case report

**DOI:** 10.1186/s42494-023-00148-x

**Published:** 2024-02-01

**Authors:** Danlei Wei, Kang Lv, Jialinzi He, Bo Xiao, Lili Long

**Affiliations:** Department of Neurology, Xiangya Hospital, Central South University, 87 Xiangya Road, Changsha, Hunan 410000 People’s Republic of China

**Keywords:** Alternating hemiplegia of childhood, *ATP1A3* gene, Mutation, Flunarizine, Case report

## Abstract

**Background:**

Alternating hemiplegia of childhood (AHC) is a rare pediatric syndrome characterized by recurring episodes of hemiplegia or quadriplegia, and frequently accompanied by dystonic posturing, choreoathetosis movements, anomalous ocular motions, and a gradual deterioration in cognitive function. The principal etiology of this disorder is traced back to mutations in the *ATP1A3* gene.

**Case presentation:**

Here, we report a 16-year-old girl with recurrent hemiplegia since her infancy. This patient has experienced paroxysmal limb weakness and aphasia for over 15 years, and has kept seeking medical attention but without receiving effective treatment. A misdiagnosis of hysteria persisted for over 4 years until the patient’s admission to our hospital. Whole-exome sequencing identified a known pathogenic heterozygous c.2270T>C (p.Leu757Pro) mutation in her *ATP1A3* gene. Notably, her clinical manifestations, including pathological emotional responses and autonomic dysfunction, differed from the established profile associated with the same *ATP1A3* mutation, which typically present with intellectual disability, a rostrocaudal symptom gradient, choreoathetosis, and dysarthria. The patient was finally diagnosed with AHC and treated with flunarizine thus significantly ameliorated hemiplegic episodes.

**Conclusions:**

This case enhances our understanding of the intricate clinical manifestations of AHC, which require careful differentiation from various diseases such as epilepsy, hysteria, and paroxysmal dyskinesias. In the diagnosis of patients presenting with suspected symptoms, adhering to a systematic approach for localizing and diagnosing neurological disorders is crucial to prevent misdiagnosis and inappropriate treatments. Additionally, when AHC is suspected in a patient, genetic testing should be considered as part of the diagnostic approach.

## Background

Alternating hemiplegia of childhood (AHC, MIM 614820) is a rare neurological disorder marked by onset of recurring episodes of hemiplegia or quadriplegia during infancy. In addition to these primary symptoms, individuals with AHC commonly exhibit accompanying manifestations such as dystonia, autonomic dysfunction, abnormal ocular movements (including monocular nystagmus, deconjugated gaze, ocular deviation, and up-rolling of the eyes), ataxia, chorea, choreoathetosis, developmental delay, and progressive cognitive impairment [1]. The prevalence of AHC is estimated to be approximately 1: 100,000 in children. While the majority of AHC cases are sporadic [2], familial occurrences have been documented, suggesting an autosomal dominant inheritance pattern.

The *ATP1A3* gene (MIM 182350), encoding the alpha-3 catalytic subunit of the Na^+^/K ^+^-ATPase transmembrane ion pump, has been established as a primary genetic contributor in AHC patients. The transmembrane ion pump comprises five extracellular domains, ten helical domains, and six cytoplasmic domains [3]. The ATP1A3 isoform is exclusively expressed in neurons of various brain regions, including the hippocampus, basal ganglia, and cerebellum. Mutations in the *ATP1A3* gene have also been identified in the patients with other conditions such as cerebellar ataxia, areflexia, pes cavus, optic atrophy, and sensorineural hearing loss syndrome (CAPOS, MIM 601338) and dystonia-12 (rapid-onset dystonia-parkinsonism, DYT12, MIM 128235) [4–6]. Therefore, detecting pathogenic mutations in patients is of great significance in diagnosis and targeted treatment.

Herein, we report an AHC patient with a known mutation c.2270T>C (p.Leu757Pro) in the *ATP1A3* gene. After being treated with flunarizine, a notable decrease was observed in both the frequency and duration of hemiplegic episodes. It is worth noting that the symptoms presented in the patient such as atypical limb weakness and episodes of suspected emotional crying and silence, which are different from the typical symptomatology of AHC and similar to the characteristics of epilepsy and hysteria. Consequently, the patient was initially misdiagnosed with epilepsy and later with hysteria until the age of 16.

## Case presentation

**Fig. 1:**
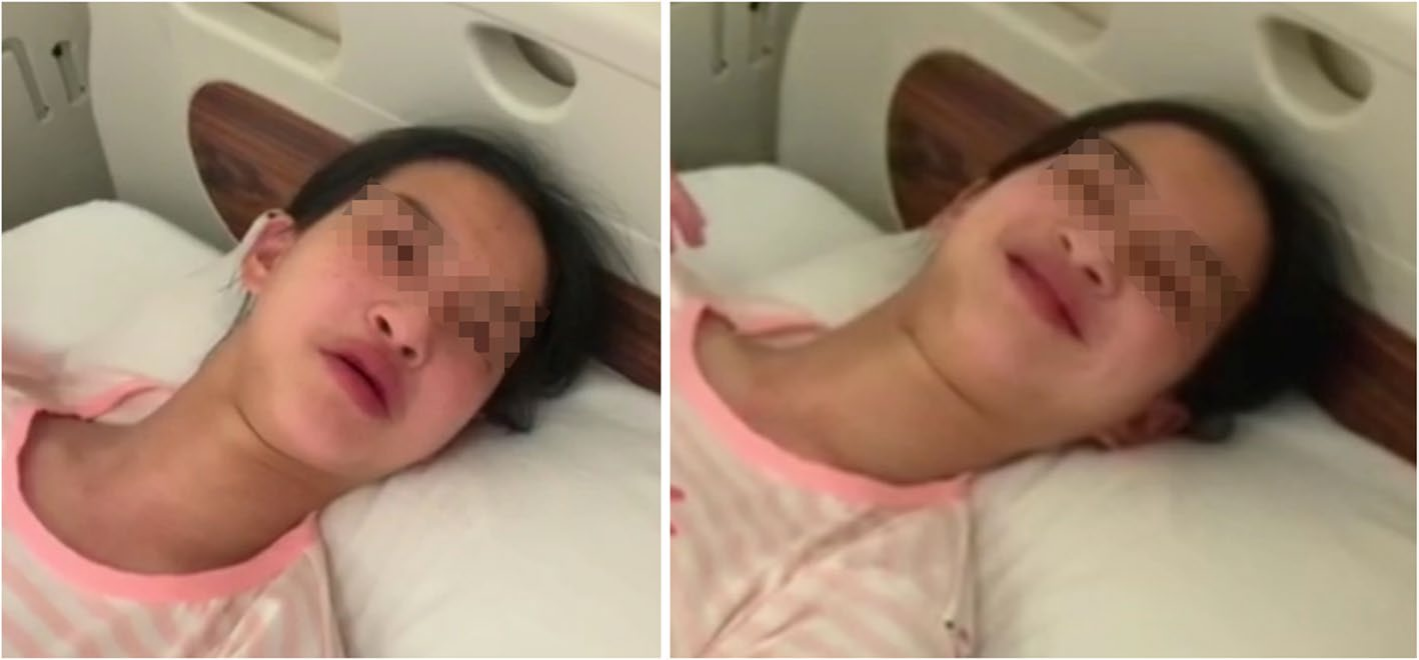
The phenotype of the patient at onset of a hemiplegic episode, which was accompanied by pathological laughing and crying

A 16-year-old girl was admitted to our outpatient clinic in December 2019 on account of a 15-year history of paroxysmal limb weakness and aphasia. During the initial few months after birth, she developed stereotyped and recurrent quadriplegia with more pronounced weakness in her left limb, which was accompanied by symptoms such as sweating, pathological laughing and crying, pupil dilation, and significant rise in blood pressure (Fig. 1) that lasted hours to three days and occurred every 4 to 15 days. The patient started walking independently at the age of 2, but her motor development was delayed, manifested as poor memory and learning abilities compared to her peers. However, there were no significant differences in physical development until the age of 12. From the age of 2 to 12, the patient continued to experience intermittent limb weakness, speech difficulties, and dragging movements in the left lower limb during episodes. In severe cases, she was unable to walk, and accompanied by sweating, chest discomfort, dizziness, and occasional forced crying and laughing, just like her previous episodes. At first, she was considered to have epilepsy at a local hospital due to the stereotypical, episodic, and repetitive features of her symptoms, but the results of the diagnostic evaluation including multiple 24-hour electroencephalograms (EEG) and brain magnetic resonance imaging (MRI) were negative. Consequently, she was highly suspected to have hysteria at the age of 12 and did not receive any special treatment over a decade. Upon admission to our hospital, the patient exhibited introverted behavior and a prospensity for irritability. Episodes occurred both during mood fluctuations and in calm circumstances as the patient grew older. These episodes were characterized by muscle weakness, speech impairment, and dragging of the left leg while walking. In severe attacks, the patient experienced an inability to walk, accompanied by chest discomfort, dizziness, and profuse sweating. Blood pressure measurements during these episodes reached as high as 180/120 mmHg. Furthermore, the patient sometimes burst into tears or laughter, often accompanied by apparent barriers to consciousness, muscular rigidity, noticeable cognitive impairment, and developmental malformation. The duration of these episodes ranged from 1 to 3 days with fluctuating symptoms. The frequency of attacks varied between intervals of 4 to 15 days. The patient could recover spontaneously, just like a normal person in convalescent periods without abnormal eyesight, audition, and olfaction. Throughout the course of the disease, the patient’s mental state, appetite, sleep patterns, urine, bowel habits and weight remained unchanged.

**Fig. 2:**
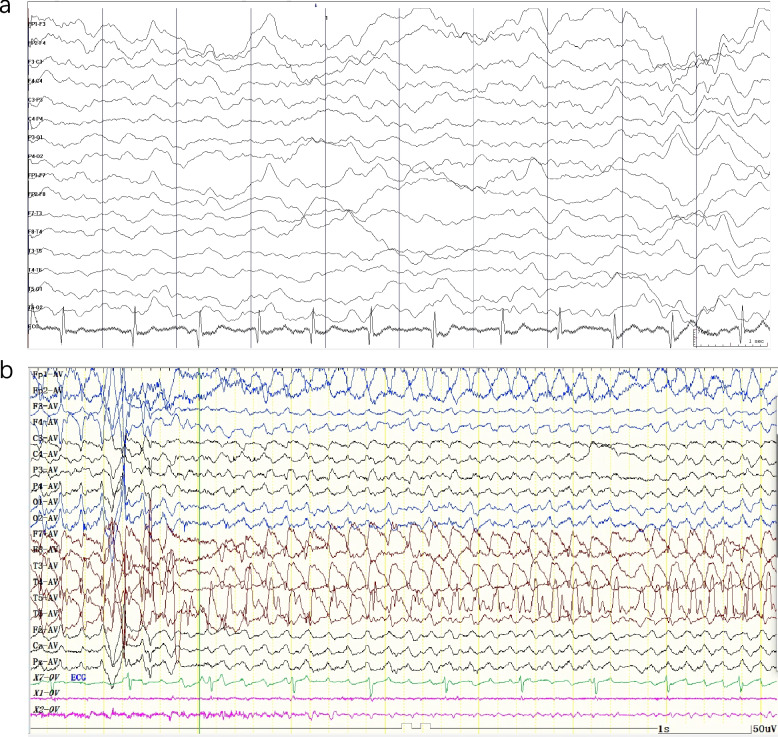
Electroencephalogram (EEG) recordings of the patient. **a** During the hemiplegic attack, interictal EEG showed basic rhythm with slow waves and poor symmetry. There were also a few single sharp waves observed in each brain area on both sides, with a greater prominence on the left side. **b** During an attack of hemiplegic episode. In the borderline adolescents, there is an increase in theta waves in the frontal regions of both hemispheres during wakefulness, and there is no abnormal discharge of visual events was observed

During episodes, medical examinations revealed bilaterally dilated pupils that responded to light. The affected limbs exhibited a muscle strength of grade 4 with normal muscle tone. Despite experiencing autonomic disturbance, the patient did not present with nystagmus. There was no obvious abnormality in the laboratory tests such as thyroid function, electrolytes, liver, and kidney function. Interictal EEG recordings during hemiplegic attacks indicated a suboptimal baseline rhythm with slow waves and poor symmetry. Besides, a few isolated sharp waves were observed bilaterally, with greater prominence on the left side. In the borderline adolescents, increased theta waves were noted in the frontal region during wakefulness, and no abnormal discharge of visual events was detected (Fig. 2).

**Fig. 3:**
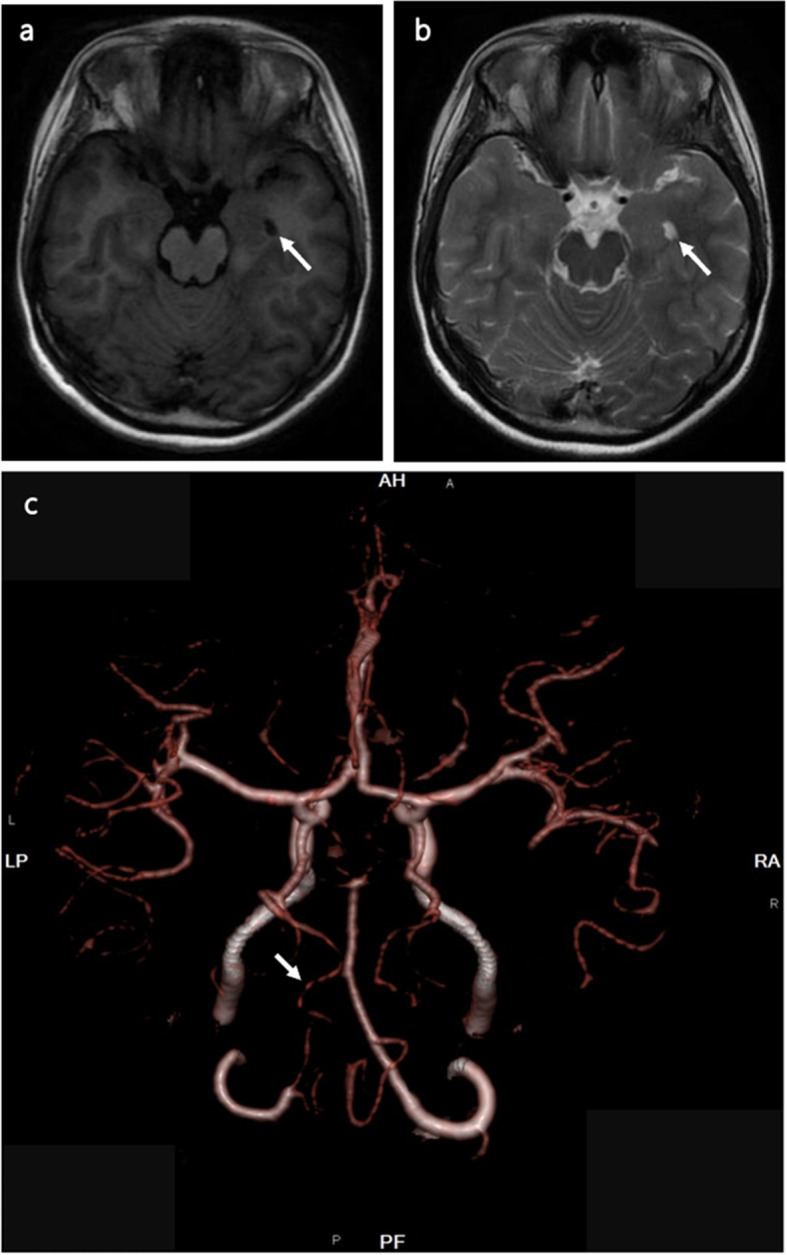
Brain magnetic resonance imaging (MRI) of the patient. **a** Dilation of the left ventricle on axial T1-weighted imaging (T1-WI). **b** Dilation of the left ventricle on axial T2-WI. **c** Cerebral CTA examination revealed multiple calcifications in the bilateral vertebral arteries and the V4 segment of the left vertebral artery was significantly thinner

Further imaging investigations like cranial MRI, susceptibility-weighted imaging (SWI), diffusion-weighted imaging (DWI), and cerebral CTA examination, did not reveal significant abnormalities associated with her clinical manifestations (Fig. 3). Mild cognitive impairment was indicated by her mini-mental state examination (MMSE) and Montreal Cognitive Assessment (MoCA) exam scores, which were 21/30 and 16/30, respectively.

**Fig. 4:**
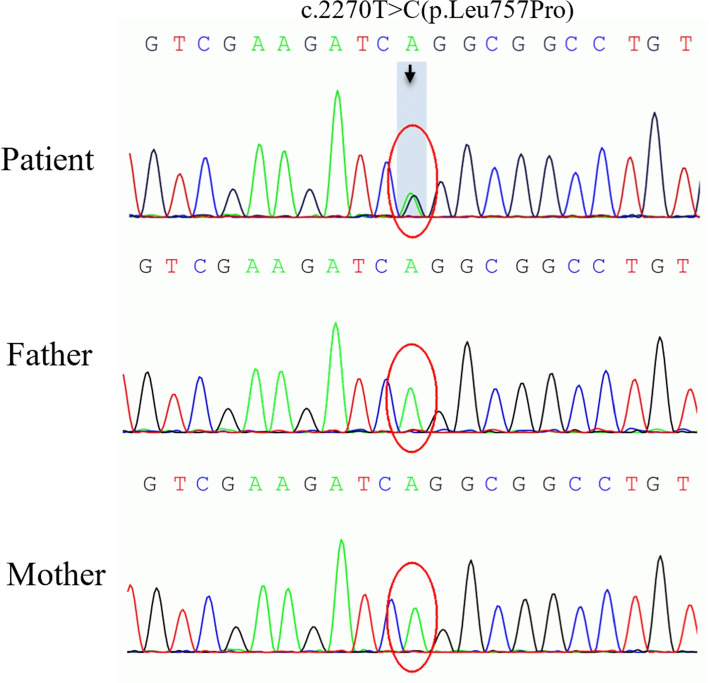
The sequencing results of *ATP1A3* gene of the patient and her parents. A heterozygous mutation in *ATP1A3* (c.2270T>C) was detected in the patient, while her parents had no variants

The patient was born in a non-consanguineous family with no history of epilepsy, migraine, or Parkinson’s disease. Informed written consent and consent for publication were obtained from the patient's parents, and this study was approved by the Ethics Committee of Central South University XiangYa Hospital. In February 2020, the whole exome sequencing was conducted and it confirmed the presence of a heterozygous p.Leu757Pro variant in exon17 of the *ATP1A3* gene (NM_152296.4, c.2270T>C) (Fig. 4) in the patient. Neither of her parents carried this mutation, indicating that it was a spontaneous occurrence.

Treatment was initiated with a dosage of 10 mg of flunarizine administered twice daily, starting in December 2019 and continuing until June 2020. During this period, an episode occurred in March 2020 and was characterized by intact consciousness, pathological laughing and crying, and limb weakness. However, this episode lasted for a significantly reduced duration of 3–4 hours compared to previous occurrences. Another episode, lasting approximately 12 hours, was observed in June 2020. Notably, symptomatic improvement was noted after the patient had slept. Since June 2020, The patient has not experienced any hemiplegic episode while receiving a combination treatment of 10 mg of flunarizine and 25mg of topiramate twice daily.

## Discussion

AHC, initially described by Verret and Steele in 1971 [7], represents a rare neurodevelopmental disorder primarily caused by de novo *ATP1A3* pathogenic variants [2, 8]. The alpha-3 catalytic subunit of the Na^+^/K^+^-ATPase transmembrane ion pump [3], encoded by the *ATP1A3* gene, is responsible for maintaining the electrochemical gradient of Na^+^/K^+^ across the membrane [9–11]. This gene plays a crucial role in the sodium conjugation, osmotic regulation of various molecules, and the excitability of nerves and muscles, which may explain the etiology of AHC symptoms [12]. This estimated incidence of AHC is approximately 1 in 100,000, although the prevalence may be underestimated due to potential misdiagnosis caused by the diverse range of paroxysms associated with this disease [3, 6].

In this study, we report a case of an AHC girl who was initially misdiagnosed with hysteria but presented with paroxysmal limb weakness and inability to speak. The paroxysmal symptoms in this case are the most interesting and challenging aspect in diagnising process. When considering the underlying cause of paroxysmal muscle weakness, the localization diagnostic spectrum narrows. Potential etiologies within the realm of the cerebral cortex include seizures, migraine, transitory ischemic attacks (TIA), paroxysmal dyskinesias, and channelopathies. Epilepsy emerges as the primary diagnostic consideration due to the patient’s paroxysmal, repetitive, and stereotypical symptoms. Frontal lobe epilepsy (FLE) can account for the symptoms and the duration of symptoms aligning with Todd’s paralysis or nonconvulsive status epilepticus (NCSE) [13]. The characteristic aspects of prefrontal seizures consist of motor semiology and ictal emotional behavioral changes. An epileptogenic lesion in the posterior regions of the prefrontal cortex, for example, can lead to concurrent tonic or dystonic posturing. Seizures originating from the anterior cingulate gyrus can first manifest as changes in emotions and autonomous nervous system symptoms, such as fear, flushing, and changes in heart rate [14]. Nevertheless, negative results of EEG during episodes effectively rule out this diagnosis. Familial migraine is excluded by the absence of migraine symptoms. Paroxysmal characteristics typically do not manifest in spinal cord or peripheral nerve disorders. Although neuromuscular junction diseases like congenital myasthenia syndrome can exhibit sporadic characteristics, they tend to be progressive and do not involve episodes of forced crying or laughter. In cases where muscles and concurrent paroxysmal symptoms are implicated, hypokalemic periodic paralysis should be considered, although it becomes less likely if the patient’s blood potassium levels remain normal during episodes.

The subsequent step involves qualitative diagnosis. Firstly, distinguishing hysteria poses a challenge, however, given that the patient’s symptoms commenced several months after birth, the likelihood of hysteria is relatively low [15]. Additionally, negative brain CTA results eliminate the possibilities of TIA and Moyamoya disease. The etiological considerations predominantly revolve around metabolic and genetic factors, given the similarity of symptoms to seizures. Potential metabolic diseases manifesting with intermittent clinical symptoms include urea cycle disorders (UCDs), organic acidemias (OAs), and mitochondrial disorders such as Leigh syndrome or MELAS (mitochondrial myopathy, encephalopathy, lactic Acidosis, and stroke-like episodes) syndrome, as well as pyruvate dehydrogenase deficiency. UCDs fall within the category of inherited metabolic diseases (IMDs) which are caused by deficiencies in crucial enzymes in the urea cycle [16–18]. Classic OAs, or branched-chain organic acidemias (BCOAs), are another subset of IMDs characterized by recurrent episodes of triple signs (including acidemia, ketonuria, and hyperammonemia), resulting from a loss of function of enzymes impeding catabolism of branched-chain amino acids (BCAAs) [16]. However, the patient’s normal blood ammonia fail to meet the diagnostic criteria for either disorders.

Primary mitochondrial disease, characterized by diverse and complex clinical features, is one of the most common metabolic diseases in humans due to dysfunction in mitochondrial respiratory chain oxidative phosphorylation. For example, Leigh syndrome may present with delayed mental and motor development, motor dysfunction, limb weakness, and seizures, whereas MELAS can involve recurrent stroke-like seizures [19, 20]. Additionally, pyruvate dehydrogenase deficiency, an X-linked dominant metabolic disorder, can also contribute to Leigh syndrome [21]. However, all pertinent blood biochemical tests, including myocardial enzymes, liver enzymes, lactate, and organic acid screenings in the patient’s blood and urine, yield negative results, failing to align with the hallmarks of metabolic diseases.

Given the negative metabolic assessment, the sporadic nature of the patient’s symptoms directs attention to another important etiological category, namely genetic factors, encompassing paroxysmal movement disorders (PMDs) and channel diseases. PMDs are characterized by episodical and transient hyperkinetic movement, comprising ataxia, ballism, chorea, and dystonia [22, 23]. Channelopathies, on the other hand, show various ion channel dysfunctions stemming from defects in proteins responsible for ion pore formation or ion movement [24]. Within this context, potential considerations include myotonia congenita (chloride channels) and AHC (sodium-potassium-ATPase).

Finally, considering the localization and qualitative diagnosis, we recommend that the patient’s parents undergo genetic testing. After 15 years of misdiagnosis, the patient received a conclusive diagnosis of AHC, attributed to a documented *ATP1A3* heterozygous mutation, specifically c.2270T>C (p.Leu757Pro). This mutation was initially reported by Rosewich H in a male AHC patient, characterized by intellectual disability, a rostrocaudal gradient, choreoathetosis, and dysarthria [5, 12]. While our patient did not exhibit ocular shock, she did display pathological laughing and crying as well as autonomic nervous dysfunction (including elevated blood pressure and profuse sweating). Notably, despite sharing the same genetic mutation, the two cases presented distinct phenotypes, highlighting the heterogeneity and intricate interplay between genotypes and phenotypes.

## Conclusions

The clinical diagnosis of AHC poses challenges due to the intricate clinical manifestations, as well as the genetic heterogeneity observed in affected individuals. However, achieving a definitive diagnosis can be facilitated through meticulous history-taking, comprehensive examination, and adherence to rigorous diagnostic protocols. The crucial role of essential genetic testing becomes apparent in elucidating the underlying etiology, guiding clinical management, and facilitating genetic counseling.

## Data Availability

Not applicable.

## Abbreviations

AHC: Alternating hemiplegia of childhood
ATP1A3: ATPase Na^+^/K^+^ transporting subunit alpha 3
BCAAs: Branched-chain amino acidemias
BCOAs: Branched-chain organic acidemias
CAPOS: Cerebellar ataxia, areflexia, pes cavus, optic atrophy, and sensorineural hearing loss
CTA: Computed tomography angiography
DWI: Diffusion-weighted imaging
DYT12: Dystonia-12
EEG: Electroencephalogram
FLE: Frontal lobe epilepsy
IMDs: Inherited metabolic diseases
MELAS: Mitochondrial myopathy, encephalopathy, lactic acidosis, and stroke-like episodes
MMSE: Mini-mental state examination
MoCA: Montreal cognitive assessment
NCSE: Nonconvulsive status epilepticus
OAs: Organic acidemias
PMDs: Paroxysmal movement disorders
SWI: Susceptibility-weighted imaging
TIA: Transient ischemic attack
UCDs: Urea cycle disorders
